# Psycho-social impact of visual impairment on health-related quality of life among nursing home residents

**DOI:** 10.1186/1472-6963-14-345

**Published:** 2014-08-15

**Authors:** Mahesh Kumar Dev, Nabin Paudel, Niraj Dev Joshi, Dev Narayan Shah, Shishir Subba

**Affiliations:** Department of Ophthalmology, Institute of Medicine, B. P. Koirala Lions Center for Ophthalmic Studies, Tribhuvan University, Maharajgunj, Kathmandu Nepal; B.P Eye Foundation, Children’s Hospital for Eye Ear and Rehabilitation Services, Bhaktapur, Nepal; Department of Optometry and Vision science, The University of Auckland, Auckland, New Zealand; Central Department of Psychology, Tribhuvan University, Kathmandu, Nepal

**Keywords:** Nursing home, Quality of life, Visual impairment, SF-36, Kathmandu

## Abstract

**Background:**

Visual impairment (VI) affects physical, psychological, and emotional well-being, and social life as well. The purpose of this exploratory study was to assess the psycho-social impact of VI on health-related quality of life (HRQoL) among nursing home residents.

**Methods:**

This cross-sectional study involved 272 residents of 60 years or older residing in seven nursing homes of the Kathmandu Valley, Nepal. Comprehensive ocular examinations, including near and distance vision assessment and refractions were carried out. VI was defined as visual acuity (VA) less than 6/18 in the better eye. Residents were divided into two groups: one group did not have VI (in whom VA was greater than or equal to 6/18 in the better eye), and the other had VI (in whom VA was worse than 6/18 in the better eye).

Face-to-face interviews were conducted filling out a 36-item The Medical Outcomes Study Short-Form (SF-36) questionnaire. The SF-36 questionnaire was scored according to the scoring algorithm SF-36 subscales.

**Results:**

The mean age of residents was 74.68 ± 8.19 years (range, 60–99 years) and the majority were female (78.68%). The mean composite score of SF-36 was 46.98 ± 13.08. VI detrimentally affected scores of both the physical and the mental components, but the impact of VI was slightly greater for the physical component than that for the mental component. There was a trend towards a lower composite score as well as each subscale score of the SF-36 in participants with VI than in those without VI.

**Conclusion:**

VI has a negative effect on HRQoL. HRQoL is reduced among nursing home residents and the reduction in the HRQoL bears a positive association with VI.

## Background

The effect that ageing will have on health care in the future is profound. Ageing physiology makes the older individual more prone to diseases and less capable of recovering his or her status quo. As life expectancy continues to rise, the geriatric population is expected to grow dramatically in the coming years [1].

The prevalence of visual impairment (VI) and blindness is higher among nursing home residents than that of the same base population living outside nursing homes [2–15]. The former usually have varieties of physical and psycho-social problems.

Population ageing has become an important social issue worldwide and improving quality of life (QoL) is among the biggest challenges for health care providers [16]. Old age often brings about health problems and a decrease in functional capacity. Ageing process and multiple physical and psycho-social problems tend to affect the QoL among nursing home residents. VI is potentially distressing because of disability and fear of total vision loss associated with it [17].

QoL can be assessed with measures of health status, functional status and psychological well-being. QoL is one of the central issues in concerning care for the elderly [18–20]. Health-related quality of life (HRQoL) is considered to be the key goal for health promotion in the elderly [21]. VI detrimentally affects HRQoL [22–26] and has a significant impact on daily functioning, including social activities [27, 28]. Visual function is important for an optimal orientation in functional and social life and has effects on physical, psychological, mental and emotional well-being [2, 3, 29–33]. Impaired vision significantly reduces activities associated with participation in society and religion, mobility, recreational and daily living etc. [2–4, 29, 30]. Vision loss in later life contributes to limitations on physical activity, reduces independent mobility, causes vision impairment and falls, imbalance, entails risks of hip fracture, mortality and underlines the need for community and/or family support [2–5, 9, 31, 33].

HRQoL in relation to nursing home residents is not well understood. This study is the first kind in the context of Nepal, though such studies have been conducted abroad. There is very little information about the impact of VI on HRQoL. The purpose of this study was to assess the psycho-social impact of VI on HRQoL among nursing home residents. To the best of our knowledge, to date, no such study on impact of VI on HRQoL among nursing home residents has been done in Nepal. QoL is increasingly being recognized as a useful outcome in health and social care research.

## Methods

### Participants

This was a descriptive, cross-sectional, and institutionalized study conducted among older adults living in seven different nursing homes in the Kathmandu Valley, Nepal.

The participants included in this study were from our earlier study which primarily aimed at determining the prevalence of VI and blindness among nursing home residents [2]. There were a total of 364 residents of 60 years or older who underwent distance and near visual acuity (VA) assessment, refraction and a complete ocular examination; however, only 272 of those participants could participate in the interview. There were various reasons for our inability to include all participants from our earlier study: some of them had disabilities and diseases, such as intellectual disabilities (6), Down’s syndrome (2), Alzheimer’s disease (2), hearing impairment (8), inability to speak and listen (25), and stroke that rendered them bedridden (14), which affected their ability to provide proper responses, whereas some residents (35) were excluded as they did not consent to this study. The information on various diseases and disorders of the residents was obtained from their medical records. The results in this study are based on the 272 residents only.

The institutional review board at the Institute Of Medicine, Tribhuvan University approved the study protocol, and the study followed the tenets of the Declaration of Helsinki. For the enrollees of this study, informed consent was gained both from the administrator of the nursing homes and the residents themselves. Enrollees’ particulars, including age, sex, marital status, educational status, and ethnicity, were noted.

### Assessment

Presenting distance VA was assessed with the Snellen chart at a six-metre distance and near acuity with the Lighthouse Near Visual Acuity Card at a 25-centimetre distance. Refraction was performed by an optometrist and the best corrected distance VA was considered in the better eye. Quantification of presenting and the best corrected distance VA was expressed in the Snellen notation and the World Health Organization’s criteria of VI were followed [34]. In light of this, VA was classified as: normal (VA greater than or equal to 6/18 in the better eye), and VI (VA worse than 6/18 in the better eye). The total residents were divided into two groups: without VI and with VI. A complete anterior and posterior segment examination was carried out in all the residents by a team of optometrists and ophthalmologists.

HRQoL was assessed by conducting face-to-face interviews using a 36-item The Medical Outcomes Study Short-Form (SF-36) Questionnaire [35–38]. The SF-36 is a set of generic, coherent, and easily administered QoL measures. The SF-36 instrument yields practical, reliable, and valid information on functional health and well-being from the patient’s point of view. It is designed for use in clinical practice and research, health policy evaluations, and general population surveys. This instrument consists of eight subscales [36, 37]: physical functioning (10 items), role limitations due to physical health (4 items), bodily pain (2 items), general health (5 items), role limitations due to emotional problems (3 items), energy/vitality (4 items), mental health (5 items), social functioning (2 items). SF-36 has both the physical and mental components. It yields psychometrically-based physical and mental health summary measures. The first four subscales belong to the physical component (PC) and the latter four subscales belong to the mental component (MC) [22, 23, 36–38].

The SF-36 was translated into Nepali and back translated into English to check the consistency in meaning. Few modifications were made in questions to make it suitable for Nepalese culture. Face validity was done with bilingual subjects to ensure that both versions provided the same response with the same score.

The face-to-face interviews were conducted by the primary author himself who was working with a psychologist and who received training in research methodology and questionnaire administration. Few other assistants were trained by the author to help him in administering the questionnaire. The agreement in the score values was compared between the interviewers randomly. There was a high agreement between the scores obtained from different interviewers. Cronbach’s Alpha of the sample of eight subscales score of the SF-36 was 0.804 which states good internal consistency and reliability of test scores.

The SF-36 was scored according to the scoring algorithm SF-36 subscales [36–38] on a scale ranging from 0 to 100. The responses to questions within each dimension are summed and transformed to generate dimension scores from 0 to 100, with 0 indicating the lowest level of function (representing severe disability) and 100 indicating the highest level of function (representing no disability) and 50 indicating the average score. A high score indicates high QoL and a low score indicates poor QoL.

Recorded data were analyzed by Statistical Package for Social Sciences (SPSS) version 17.0 and Microsoft Excel version 2010. Appropriate statistical tools were implemented depending upon the distribution of the variables.

## Results

### Demographics of study participants

Table 1 presents demographic characteristics. The mean age of the residents was 74.68 ± 8.19 years (range, 60–99 years). The majority of residents (43.38%, 118) were in the 70–79 years age group. There was no statistically significant difference between the mean age of male (75.07 ± 7.99 years) and female residents (74.58 ± 8.27 years) (t-test, p = 0.687). The vast majority (78.68%, 214) were female residents, and most were widowed (63.18%).

**Table 1:** **Demographics of the study enrollees (based on presenting distance VA)**

Characteristics	Without VI, N (%)	With VI, N (%)
	75 (27.58)	197 (72.42)
**Age Range (years**)		
60-69	25(33.33)	45(22.84)
70-79	38(50.67)	80(40.61)
80-89	12(16.00)	66(33.50)
90-99	-	6(3.05)
**Sex**		
Male	23(30.67)	35(17.77)
Female	52(69.33)	162(82.23)
**Race/Ethnicity**		
Upper caste	25(33.33)	71(36.04)
Advantaged Janjatis	42(56.01)	98(49.75)
Non Dalit Terai	1(1.33)	1(0.51)
Disadvantaged Janjatis	7(9.33)	24(12.18)
Dalit	-	3(1.52)
**Education**		
Illiterate	61(81.33)	165(83.76)
Simple read and write	12(16.01)	30(15.22)
Primary school	1(1.33)	1(0.51)
Secondary school	1(1.33)	1(0.51)

VA: Visual acuity, VI: Visual impairment.

**Note**: The percentage values in parentheses are by considering numbers in each subgroup as 100%.

### Near and distance acuity and visual impairment

Mean presenting near acuity was 14 N ± 2.28 and the best near acuity after appropriate near correction was 8 N ± 1.61. The difference was statistically significant (Wilcoxon Signed Ranks Test, p = 0.042).

Considering the presenting distance VA worse than 6/18 in the better eye, 197 (72.42%) residents had VI, and considering the best refractive correction, 123(45.22%) residents had VI. Refractive correctable VI in our participants was 27.20%.

Cataract was the leading cause of non-refractive VI and blindness, which was followed by age-related macular degeneration, corneal opacity, glaucoma and macular scar.

### Health-related quality of life (HRQoL)

The mean overall (composite) score of the SF-36 was 46.98 ± 13.08. HRQoL was deleteriously reduced among nursing home residents. The overall mean physical component score (PCS) was 48.95 ± 16.39 and the mean mental component score (MCS) was 45.09 ± 12.20 (Table 2). This difference between PCS and MCS was statistically significant (Independent t-test, p = 0.02).

**Table 2:** **Mean score of each subscale, composite and components of SF-36 questionnaire**

SF-36 indicators	Mean score (SD)	Without VI (SD)	With VI (SD)	p-value
Physical functioning	54.14 (20.10)	62.71(20.02)	50.87(19.20)	0.00*
Role limitation: PH	46.05(33.26)	58.41 (32.28)	41.34 (32.49)	0.00*
Bodily Pain	44.34 (19.57)	47.00 (16.64)	43.32 (20.52)	0.16
General health	51.28(16.65)	57.14 (16.30)	49.05 (16.27)	0.00*
Vitality	46.35(10.85)	47.82 (8.89)	45.79 (11.48)	0.16
Social functioning	44.09(18.44)	42.59 (16.44)	44.66 (19.15)	0.40
Role limitation: EP	41.78 (31.73)	50.48 (32.844)	38.46 (30.75)	0.05
Mental health	48.15 (11.10)	48.94 (10.67)	47.86(11.27)	0.47
**Composite SF-36**	**46.98 (13.08)**	**51.88 (12.28)**	**45.11 (12.91)**	0.00*
**Physical component**	**48.95(16.39)**	**56.31(14.74)**	**46.15(16.16)**	0.00*
**Mental component**	**45.09(12.20)**	**47.46 (12.27)**	**44.19 (12.08)**	0.04*

SF: Short-form health survey, PH: Physical health, EP: Emotional problem, VI: Visual impairment.

**Note**: *Indicates significant differences in the scores.

There was a trend towards lower SF-36 composite scores in participants with VI than in those without VI (Table 2). VI has a negative impact on HRQoL among nursing home residents and this was statistically significant (Independent t-test, p = 0.00; 95% CI of the difference 3.37–10.18). This shows poorer HRQoL of life among VI group than in those without VI.

Participants with VI had associated decrement in scores on all subscales except for the social functioning subscale; there was a consistent deterioration in scores of SF-36 subscales in participants with VI than in those without VI (Table 2). Three domains of the PC: physical functioning, role limitations due to physical health and general health had been more affected by VI than any others (Table 2).

VI detrimentally affected both the PCS and the MCS. The difference in the PCS and the MCS between residents with VI and residents without VI was statistically significant (Independent t-test, p =0.00 for PC and p = 0.04 for MC) (Table 2). While VI detrimentally affected both the PC and the MC, the former suffered slightly greater impact.

### Gender and HRQoL

There was no statistically significant difference between the mean SF-36 scores in male (mean, 48.05 ± 11.97) and female residents (mean, 46.69 ± 13.37) (Independent t-test, p = 0.48). Thus, regarding the impact of VI on HRQoL, there was no sex predilection.

### Association between age and subscale scores of SF-36

**Figure 1:**
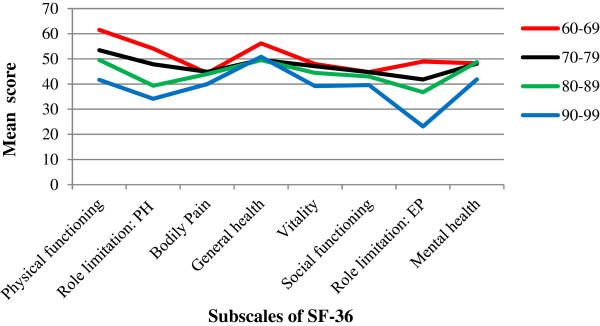
**Association between age and subscales score of SF-36.** PH: Physical health, EP: Emotional problem, VI: Visual impairment.

HRQoL was significantly associated with age (Pearson’s r = −0.22, p = 0.001). As age increased, SF-36 scores gradually reduced. Scores for all SF-36 dimensions except general health and mental health, gradually decreased with age (Figure 1).

## Discussion

This exploratory study was conducted to assess the psycho-social impact of VI on HRQoL among nursing home residents. HRQoL was significantly poor among nursing home residents. The mean composite score of the SF-36 was 46.98 ± 13.08, indicating that the overall health of nursing home residents were decreased by nearly fifty percent. The composite score of the SF-36 in our study was comparatively lower in comparison to Canbaz et al.’s [39] and Sabbah et al.’s [40] studies conducted in the elderly people who were not institutionalized and were residing outside the nursing homes. Thus, it is important to have information about the type of care needed in order to support their independence and maximize their quality of life. Hence, they are in greater need for community and family support [34].

Cataract was the leading cause of VI in our study. It could be accounted for by lack of eye care professionals who routinely serve nursing home residents. Ocular health is still not incorporated into the national health policy of the country. Cataract causes loss in subjective quality of vision regardless of the presence of other ocular co-morbidity. Cataract surgery improves the objective measurements and subjective QoL and visual functioning [41]. Hence, it is essential to create awareness that cataract surgery is life-enhancing and improves the QoL.

Our results demonstrated that the mean scores of each subscale of the SF-36 was much lower in comparison to the studies conducted by Chia EM et al. (2004) [22] and Chia EM et al. (2003) in Australia [23] (Table 3). It could be due to inadequate health care status among nursing homes of developing countries compared to that among nursing homes of developed countries. Unlike the study by Chang et al. in Taiwan [18], we observed that subscales of SF-36 scores were comparatively lower in all subscales except physical functioning and general health (Table 3).

**Table 3:** **Comparison of mean subscale scores of SF-36 from other studies**

Subscale	Present study (2012)	Chia EM et al. [23]	Chia EM et al. [22]	Chang HT et al. [18]
Physical functioning	54.14 (20.10)	73.3	72.3 (0.4)	42.34 (11.71)
Role limitation: PH	46.05(33.26)	68.7	67.0 (0.7)	48.25 (12.10)
Bodily Pain	44.34 (19.57)	70.6	70.6 (0.5)	53.15 (10.09)
General health	51.28(16.65)	68.3	68.1 (0.4)	43.11 (9.19)
Vitality	46.35(10.85)	62.4	62.0 (0.4)	56.97 (9.03)
Social functioning	44.09(18.44)	84.2	84.1 (0.4)	52.85 (8.61)
Role limitation: EP	41.78 (31.73)	81.5	81.0 (0.7)	51.69 (9.73)
Mental health	48.15 (11.10)	78.5	78.8 (0.3)	52.46 (9.07)
**Physical component**	**48.95(16.39)**	**45.4**	**45.0 (0.2)**	**NA**
**Mental component**	**45.09(12.20)**	**51.9**	**52.0 (0.2)**	**NA**

SF: Short-form health survey, PH: Physical health, EP: Emotional problem, NA: Not applicable.

Our measure includes the SF-36 which has both the PC and the MC [36–38]. The overall MCS was slightly lower than PCS in our study. This was in accordance with Yang et al.’s study [42] conducted among Chinese caregivers of the older adults living in the community but was in contrast to the studies conducted by Elliot AF et al.(2009) [5], Chia EM et al. (2004) [22] and Chia EM et al. (2003) [23] (Table 3). The reason might be that residents are mentally dissatisfied due to compulsion to stay in nursing homes because of lack of appropriate care by the family members which disrupted mental QoL more than physical QoL. It could also be due to the fact that nursing home residents might be mentally weak and psychologically depressed regarding their visual problems. Nursing home residents are more prone to depression and other types of mental health problems due to impaired vision [4]. The information helps in understanding the impact of VI, offering psycho-social support and calling for eye care interventions in HRQoL among nursing home residents. This suggests a greater need for eye care services in nursing home residents. It is important that their needs not be ignored to maximize QoL.

HRQoL was reduced in both the groups of residents: without VI and with VI. It could be due to other associated co-morbid diseases such as hypertension, diabetes, stroke, angina, multiple sclerosis etc. VI deleteriously affected HRQoL of nursing home residents; however, the impact of VI was more sensitive to vision-specific QoL as shown by our earlier study [3]. HRQoL was poorer in residents with VI than in residents without VI. This was similar to Chia EM et al.’s study [22]. It could be explained on the basis that VI reduces physical activity, independent mobility, participation in social functioning, and daily living activities. The impact of VI on HRQoL may be due to poor utilization of accessible health and eye care services by nursing home residents of developing countries. Neither the government nor any concerned group has paid any interest in the provision of health and eye care services.

Our study reported that VI had an impact on both the PC and the MC of the SF-36. In contrary to Chia EM et al.’s study [22], this study demonstrated that VI had a slightly greater impact on the PC than on the MC. It could be due to residents’ perception that visual loss is definitely to occur at old age. The other inference could be that VI limits residents’ vigorous activities like running, lifting heavy objects and participating in strenuous sports and moderate activities like moving the table, climbing stairs, lifting grocery etc. which directly affects the physical domains. It might also be due to other associated co-morbid diseases like hypertension, stroke or diabetes in our participants which may affect PC more than MC. Stroke had a greater impact on physical domains and a milder impact on mental domains [22]. These could be the reasons for the three domains of the PC-physical functioning, role limitations due to physical health and general health being more affected by VI than the other subscales. The decrease in function and well-being associated with VI is integrated into a person’s HRQoL and is not easily isolated from other medical conditions [22].

The findings of this study have a vital implication because the burden of VI is gradually increasing. Should we pay more attention to the environment of nursing homes and residents’ hobbies and special interests, HRQoL can increase [43]. Besides these ways of improving HRQoL, regular visits, systematic assessment and intervention, especially focusing on their symptoms, can lead to higher HRQoL [44]. Our strategies to promote and enhance HRQoL should also include management of all co-morbid illnesses and educating them [43].

Study limitations must also be acknowledged. Much as a widely used generic measure as the SF-36 is, this tool is not a vision-specific measure. VI has more impact on the vision-specific QoL than on the HRQoL. There was no assessment of co-morbidity, so we do not appreciate whether the association between VI and SF-36 truly reflects the impact of VI or may reflect generally poorer health among those with vision problems. The non-use of Rasch analysis to score the questionnaire could be another limitation of the study. The Rasch analysis enables interval level estimates from ordinal questionnaire responses. Rasch scales are strictly one-dimensional and thus allow for unambiguous interpretation of diagnostic results. It facilitates more differentiated and clinically meaningful data interpretation and enhances analysis of clinical data. It also provides the validity of the instrument more precisely and accurately [45–49].

The strength of this study is that we attempted to explore the impact of VI on HRQoL by using the SF-36 instrument. The SF-36 health surveys are the most widely used tools in the world for measuring patient-reported outcomes [36–38]. Although generic health outcome measures, such as the SF-36 may not be so sensitive to visual health but they have the ability to allow comparison across a wide range of medical conditions [22]. They can be used across age groups, diseases, and treatment groups, and are appropriate for a wide variety of applications. Its subscales have demonstrated good internal consistency, reliability, and validity [36, 37]. To the best of our knowledge, this type of study has never been conducted in a developing country like Nepal and very few in the other developed countries. This study can assist the government and the concerned authority in formulating and carrying out policies on making appropriate interventions in nursing homes of developing countries. Recently, there is a growing recognition of the importance of patient-reported outcomes of visual functioning.

## Conclusion

HRQoL is poor among nursing home residents. VI has a significant impact on HRQoL. Significant impact of VI on HRQoL suggests greater need for eye care services in them and psycho-social, family and community support. They have no one to look after them; hence, the government and the concerned group should take the initiative in the provision of their welfare and support.

Enhancing HRQoL should be given high priority in nursing homes. The residents need help from formal caregivers to maximize coping, adjustment, and independence and heighten QoL.

Further work on whether correctable or non-correctable VI has more impact on HRQoL, is warranted. Future work also needed to assess HRQoL after correction of VI and comparing the difference before and after the intervention.
